# Calcium isotope compositions as a means to trace carbonate recycling

**DOI:** 10.1093/nsr/nwab173

**Published:** 2021-09-11

**Authors:** Shichun Huang, Stein B Jacobsen

**Affiliations:** Department of Geoscience, University of Nevada, Las Vegas, USA; Department of Earth and Planetary Sciences, Harvard University, USA

## Abstract

Marine carbonate, an important CO2 reservoir, is continuously sent to the Earth's deep interior at subduction zones, forming an essential part of the global carbon cycle. The pros and cons of using calcium isotope compositions to trace marine carbonates recycled into the mantle are discussed in this Perspective.

Marine carbonates, such as sedimentary carbonates and secondary carbonates formed during marine alteration processes, are returned to the mantle at subduction zones, and their fates regulate the global carbon cycle. Some carbonates are destroyed at the subduction zones, contributing to arc volcanism [1,2]. However, some subducted carbonates may be sampled by intraplate volcanism via mantle plumes or upwelling in big mantle wedges or rifts (e.g. [3,4]). Geochemists are exploring novel geochemical and isotopic tools that can be used to trace the recycled carbonate signatures in both mantle derived rocks (basalts) and mantle rocks (peridotites; see contributions in this issue).

Marine carbonates are mostly CaCO_3_ with ∼40% CaO while the Earth's mantle typically has only 3.5% CaO. If subducted carbonates have a different Ca isotope composition, measured as δ^44/40^Ca (= [(^44^Ca/^40^Ca)_sample_/(^44^Ca/^40^Ca)_standard_ – 1] × 1000), compared to the mantle [Fig. 1], then δ^44/40^Ca can be a ‘smoking gun’ for tracing recycled carbonates. This idea led Huang *et al.* [3] to use δ^44/40^Ca to estimate the contribution of recycled carbonate in the mantle source of Hawaiian shield lavas.

**Figure 1.**
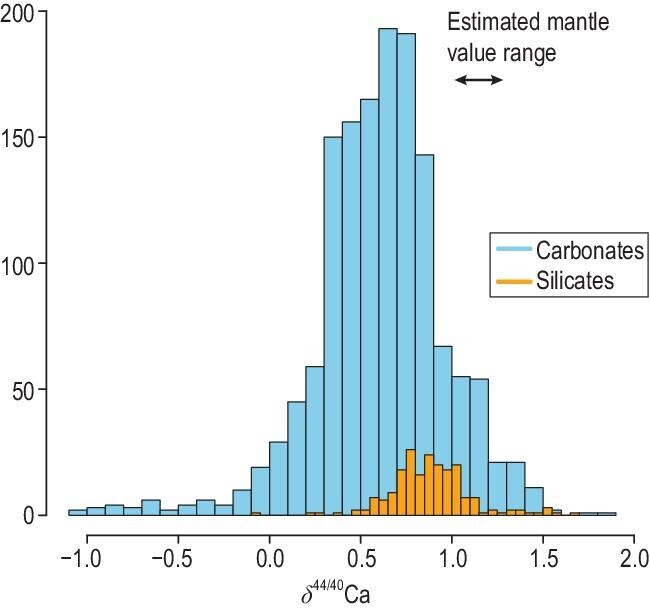
Histogram of δ^44/40^Ca in carbonates and silicates. Although carbonates have a lower average than silicates (0.61 ± 0.36 [1σ] vs. 0.89 ± 0.22 [1σ]), the δ^44/40^Ca distributions in carbonates and silicates largely overlap. The estimated mantle values range from 0.94 to 1.05 (see Antonelli and Simon [9] for a summary), which are close to the average silicate value. We note that the highest and the lowest δ^44/40^Ca in silicates are found in mantle rocks: United States Geological Survey (USGS) standard samples DTS-1, -2 (Twin Sisters dunite) and Fe-rich peridotitic xenoliths from the North China Craton [14], respectively. As a consequence, the average silicate δ^44/40^Ca value is close to, if not representative of, mantle value. Data are from the compilations in refs [11] and [16].

Recently, Amsellem *et al.* [5] reported low δ^44/40^Ca values (∼0.6 lower than the mantle value) for a global sampling of carbonatites (up to 3 Ga). They argued that the low δ^44/40^Ca reflects contributions from surface carbonates recycled into the mantle sources of these carbonatites. However, a subsequent study found that global carbonatites, after screening for alteration, have the same δ^44/40^Ca range as that shown in silicates [6]. This result is consistent with the finding that peridotites metasomatized by carbonate-rich melt have δ^44/40^Ca similar to that in unmetasomatized peridotites [7]. In contrast, Banerjee *et al.* [8] found that the majority of ancient carbonatites (>300 Ma) have mantle-like δ^44/40^Ca, but some young carbonatites (<300 Ma) have significantly low δ^44/40^Ca value (∼0.5 lower than mantle value).

Both carbonates and silicates have large δ^44/40^Ca variations, and they largely overlap with each other (Fig. 1). The mantle value has been estimated based on δ^44/40^Ca in mid-ocean ridge basalts (MORBs), peridotites and chondrites, ranging from 0.94 to 1.05 (see Antonelli and Simon [9] for a summary). This value is close to the average of silicates, 0.89 ± 0.22 (1σ, Fig. 1). It is evident that recycled carbonates can have higher, similar or lower δ^44/40^Ca compared to the mantle. If the subducted carbonates have mantle-like δ^44/40^Ca values, Ca isotope composition cannot distinguish between carbonate and mantle contributions. That is, δ^44/40^Ca cannot be used to trace carbonate recycling in this case. However, it is unlikely that all subducted carbonates have mantle-like δ^44/40^Ca values, and δ^44/40^Ca can be used with other geochemical and isotopic parameters to trace recycled carbonates. This is the approach that we used to identify recycled carbonate in the Hawaiian mantle plume. Specifically, Huang *et al.* [3] found ∼0.3 variation in δ^44/40^Ca in a group of Hawaiian shield lavas. The δ^44/40^Ca variation is correlated with Sr/Nb and ^87^Sr/^86^Sr, which are also tracers of marine carbonates. These correlations are best explained by adding up to 4% recycled carbonate into the Hawaiian mantle plume. In this case, the other two carbonate-sensitive parameters, Sr/Nb and ^87^Sr/^86^Sr, are used to constrain the δ^44/40^Ca characteristics of the recycled carbonate sampled by Hawaiian mantle plume. However, the contribution of recycled carbonate estimated using only Sr/Nb and ^87^Sr/^86^Sr relies on the assumed concentrations of Sr and Nb in the recycled carbonates, which can vary up to several magnitudes. The relative contribution of recycled carbonate is best constrained by δ^44/40^Ca using mass balance, because Ca is a major element in carbonates and its content does not vary too much.

Although δ^44/40^Ca has the potential to trace carbonate recycling, several issues must be better addressed/understood before making full use of δ^44/40^Ca. First, there are considerable inter-laboratory differences among published δ^44/40^Ca data. For example, published δ^44/40^Ca values in the BHVO-1 and BHVO-2 standards range from 0.73 to 0.96 [10], much larger than the typical analytical uncertainty of ±0.05. As discussed above, three recent studies on global carbonatites [5,6,8] yielded very different results. Sun *et al.* [6] suggested that the lower δ^44/40^Ca in Amsellem *et al.* [5] might result from the matrix effect of rare-earth elements (REEs) that may not be effectively removed during their chemical separation of Ca. However, not all carbonatites studied by Amsellem *et al.* [5] have high REE contents, and their measured carbonatite δ^44/40^Ca does not correlate with REE content. Therefore, the exact reason for such large inter-laboratory difference remains unclear. Nevertheless, the reported large inter-laboratory difference in published Ca isotope studies must be understood and addressed before making full use of δ^44/40^Ca data to trace recycled carbonates.

Second, partial melting effects on δ^44/40^Ca should be better addressed. Using a combination of δ^44/40^Ca measurements and *ab initio* calculation results, Zhang *et al.* [11] showed that partial melting of spinel peridotite introduces only a minor effect on δ^44/40^Ca in melts, which is smaller than typical analytical uncertainty. Instead, a large δ^44/40^Ca effect, of up to 0.3, can be produced in melting residues. Wang *et al.* [12] found that garnets have higher δ^44/40^Ca, by 0.1 to 0.4, than their coexisting clinopyroxenes in a series of eclogites and garnet-peridotites from the Dabie-Sulu orogen, China, consistent with the *ab initio* calculation results [13]. Wang *et al.* [12] also inferred that jadeite-rich clinopyroxene, which is typically stable under high pressure, tends to be enriched in heavy Ca isotopes. Consequently, it is expected that partial melts of garnet peridotites and garnet pyroxenites have lower δ^44/40^Ca, as observed in low-Mg adakitic rocks [12]. However, the exact δ^44/40^Ca effects during partial melting of garnet-bearing lithology are not well constrained. This is because there are no available mineral-melt Ca isotope fractionation factors under high pressures (>3 Ga) within the garnet stability field. The low-pressure mineral-melt Ca isotope fractionation factors inferred by Zhang *et al.* [11] should not be directly applied to higher pressures, because the structures of minerals and melt respond very differently to pressure. Similarly, Ca isotopic effects during partial dissolution and breakdown of carbonates, and isotopic exchange among carbonate and silica minerals at subduction zones, are also not well constrained due to the lack of reliable mineral-fluid Ca isotope fractionation factors. Mineral-melt/fluid Ca isotope fractionation factors under high pressures must be determined experimentally and/or by *ab initio* calculations before making full use of δ^44/40^Ca data to trace recycled carbonates.

Last, kinetic processes can also fractionate Ca isotopes. Zhao *et al.* [14] found very low δ^44/40^Ca, down to −0.1, in some peridotite xenoliths from the North China Craton, and they attributed the low δ^44/40^Ca to the melt-rock reaction that occurred at the base of the lithospheric mantle. Specifically, lighter Ca isotopes are preferentially incorporated into clinopyroxene during the melt-mantle reaction. Antonelli *et al.* [15] reported low δ^44/40^Ca in both natural and experimentally grown plagioclases, and they attributed the low δ^44/40^Ca in plagioclases to kinetic effects during magmatic plagioclase crystallization. Additional geochemical and isotopic tracers must be used together with δ^44/40^Ca to distinguish between recycled carbonates, metasomatized mantle and plagioclase cumulates.

In summary, because of the large δ^44/40^Ca variation in carbonates compared to the mantle, and the large CaO concentration difference between marine carbonates and the mantle, Ca isotope composition is a potentially powerful tool for tracing the recycling of carbonates into the mantle, especially when used in combination with other geochemical and isotopic tracers for carbonates (see contributions in this Special Topic). However, there remain some issues that should be addressed: (i) large inter-laboratory differences in Ca isotopic measurements, and (ii) a better understanding of Ca isotopic effects during igneous, metasomatic and metamorphic processes, including under high pressures, using analytical, experimental and theoretical approaches.
